# Somatostatin Receptors and Chemokine Receptor CXCR4 in Lymphomas: A Histopathological Review of Six Lymphoma Subtypes

**DOI:** 10.3389/fonc.2021.710900

**Published:** 2021-07-08

**Authors:** Tiina Juntikka, Samuli Vaittinen, Tero Vahlberg, Sirkku Jyrkkiö, Heikki Minn

**Affiliations:** ^1^ Department of Oncology and Radiotherapy, Turku University Hospital, University of Turku, Turku, Finland; ^2^ Department of Pathology, Turku University Hospital, University of Turku, Turku, Finland; ^3^ Department of Clinical Medicine, Biostatistics, University of Turku, Turku, Finland

**Keywords:** lymphoma, somatostatin receptor, SSTR, chemokine receptor 4, CXCR4, immunohistochemistry

## Abstract

**Background:**

Somatostatin receptors (SSTR) and chemokine receptor CXCR4 are expressed in lymphomas, while the abundance is known to be heterogeneous in different subtypes of lymphomas. Targeting tumor cells expressing these receptors might add to therapeutic opportunities while radiolabeled ligands for both imaging and therapy have been developed. The aim of this study was to establish SSTR subtype 2, 3 and 5 and also CXCR4 status immunohistochemically in six different lymphoma subtypes: diffuse large B-cell lymphoma (DLBCL), follicular lymphoma (FL), mantle cell lymphoma (MCL), mucosa-associated marginal B-cell lymphoma (MALT), Hodgkin lymphoma (HL) and peripheral T-cell lymphoma (PTCL).

**Material and Methods:**

This study included a total of 103 lymphoma patients (24 DLBCL, 22 FL, 18 HL, 9 MALT, 20 MCL and 10 PTCL) diagnosed in the Southwest hospital district of Finland during 2010-2019. SSTR 2, 3 and 5 and CXCR4 expression was analyzed immunohistochemically (IHC) in lymphoma samples obtained from local archival Biobank tissue repository. Immunopositivity of each receptor was scored on a four-point scale accounting for staining intensity and proportion of positively stained tumor cells.

**Results:**

Of different SSTR subtypes SSTR2 immunopositivity was most common and seen predominantly at the cell membrane of the malignant cells in 46-56% of DLBCL, HL and FL. CXCR4 co-expression was frequently present in these cases. SSTR3 and SSTR5 IHC were negative in DLBCL and FL but in HL SSTR expression was more heterogenous and SSTR3 and SSTR5 positivity was found in cytoplasm in 35% and 25% of cases. 2/4 blastoid MCL variants and one pleomorphic MCL variant had positive CXCR4 IHC whilst all other MCL cases (85%) were negative for all receptors. 30% (n=3) of the PTCL patients had positive SSTR5 IHC and CXCR4. MALT lymphomas were negative for all receptors.

**Conclusion:**

SSTR2 and CXCR4 are found in DLBCL, FL and HL and co-expression of these receptors is common. Although in general expression of SSTRs and CXCR4 is heterogenous and very low in some subtypes such as MCL and MALT there are also patients with abundant expression. The latter are candidates for trials studying SSTR2 and/or CXCR4 based treatments in the future.

## Introduction

Somatostatin receptors (SSTRs) are expressed in lymphomas (1) but generally at lower level compared to neuroendocrine tumors (NETs) where SSTR-based imaging (PET/CT with ^68^Ga-DOTA-peptides) and SSTR-based treatments (^117^Lu-DOTATE radiotherapy or subcutaneous Octreotide) are routinely used (2–4). SSTR-positive lymphomas represent a potential pitfall in PET/CT with ^68^Ga-DOTA-peptides (5–8) while clinical significance of SSTR expression in lymphomas remains elusive (9–12). However, lymphomas are highly radiation sensitive (13) and use of SSTR-based peptide receptor radionuclide therapy (PRRT) might deserve attention in management of selected cases.

Chemokines are important for regulation of immune cell development and migration. Specifically, chemokine receptor CXCR4 along with its ligand stromal-derived factor-1 (CXCL12) is involved in signaling pathways of several hematologic malignancies including lymphomas (14). CXCR4 immunohistochemistry (IHC) has been shown to be highly positive e.g. in mucosa-associated lymphoid tissue (MALT) type lymphomas (15) and CXCR4 antagonists such as plerixafor prevent disease progression in non-Hodgkin lymphoma *in vitro* especially when combined with rituximab (16–18). In line with SSTRs it is possible to target CXCR4 for radionuclide imaging and treatment while it is likely that strong receptor expression is mandatory for a successful response to PRRT and non-radioactive therapeutic approaches.

To our best knowledge, there are no previous immunohistochemical studies where SSTR and CXCR4 statuses have been analyzed in several lymphoma subtypes simultaneously. With the encouragement given by previous data on expression of SSTR and CXCR4 in a variety of lymphomas we determined SSTR 2, 3 and 5 and chemokine receptor CXCR4 expression immunohistochemically from tissue samples archived recently in local biobank. We obtained samples from 103 patients representing six different lymphoma subtypes. Our aim was to evaluate whether consistent patterns of receptor expression could be found with potential to assist in selection of candidates for SSTR or CXCR4 based treatment methods in the future.

## Material and Methods

### Patients

A total of 103 patients were included in this retrospective study. Inclusion criteria were histologically verified lymphoma diagnosis of DLBCL, FL, MCL, MALT, HL or PTCL/ALCL; age over 18 years; paraffin-embedded tumor samples with IHC stainings available from local university-based biobank; and an informed consent. New tissue samples were not collected nor did the patients need to undergo any additional examinations or hospital visits.

Tissue samples were excisional (or biopsied) lymph nodes (63%), bone marrow trephines (7%) or biopsies from extranodal sites (30%). Biopsies were mainly diagnostic (n=88) or as in few cases, taken at a relapse or progression (n=12). Additionally, three tissue biopsies were taken from a transformed disease (HL patients nos. 51, 58 and 61). Patient characteristics are presented in **Table 1**.

**Table 1:** Patients characteristics.

	n (%)
**Patients**	103
**Males**	57 (55%)
**Females**	46 (45%)
**Mean age (range)**	63 (20-86)
**Stage**	
**I-II**	28 (27%)
**III-IV**	75 (73%)
**DLBCL**	24 (23%)
**FL**	22 (21%)
**MCL**	20 (19%)
**MALT**	9 (9%)
**PTCL**	10 (10%)
**HL**	18 (18%)

### Immunohistochemistry (IHC)

SSTR subtypes 2, 3 and 5 and chemokine receptor CXCR4 immunohistochemical stainings were performed to the patients’ formalin-fixed paraffin-embedded tumor samples sectioned at 3 μm by using commercial rabbit monoclonal antibodies SSTR2/UMB1 (dilution 1:500), SSTR3/UMB5 (dilution 1:500), SSTR5/UMB4 (dilution 1:50 or 1:500) and CXCR4/UMB2 (dilution 1:500) (Abcam, Cambridge, UK). Staining was done with Labvision Autostainer 480S and Orion 2 steps detection system goat anti ms/rb HRP WellMed T100HRP was used as a secondary antibody. Human pancreas was used as a control tissue.

**Figure 1:**
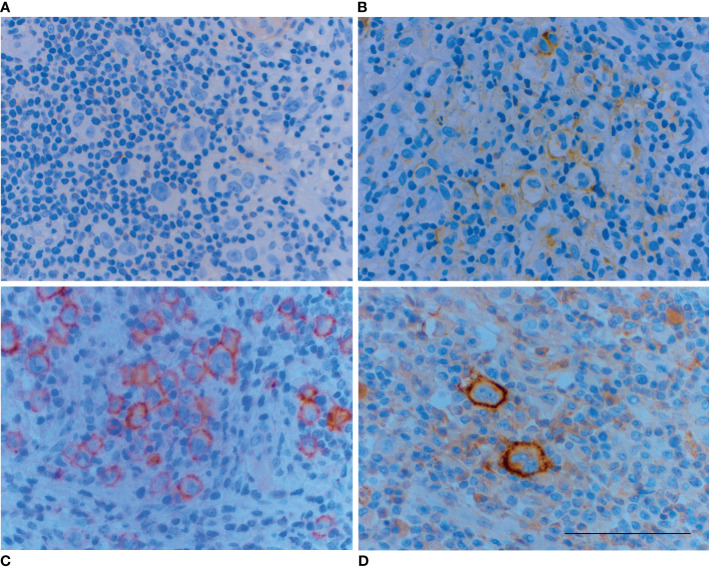
Examples of different staining intensities on the cell membrane of malignant Reed-Sternberg and Hodgkin cells: **(A)** no staining (score 0), **(B)** mild staining (score 1), **(C)** moderate staining (score 2) and **(D)** strong staining (score 3). Brown color indicates positive staining. Scale bar=100 µm.

An experienced lymphoma pathologist analyzed IHC results and categorized samples visually by intensity of staining in malignant cells: 0 (no staining), 1 (mild staining), 2 (moderate staining) and 3 (strong staining). (**Figure 1**) If the IHC revealed positive staining (1–3), also a percentual proportion (0-100%) of the positively stained malignant cells was determined. A four-point scale was then developed to further describe the receptor expression (**Table 2**).

**Table 2:** Four-point scale used in scoring IHC results.

Score	Expression	Definition
**0**	negative	no staining
**1**	mild	mild staining<75% or moderate staining in <25%
**2**	moderate	mild staining in >75%, moderate staining >25% or strong staining in <25%
**3**	strong	moderate staining in >75% or strong staining >25%

### Statistical Analyses

Descriptive statistics is presented as mean (range) for age and frequencies (percentages) for categorical variables. Statistical analyses were performed using IBM SPSS Statistics for Windows, version 26. (IBM Corp., Armonk, NY).

## Results

### Diffuse Large B-cell Lymphoma

**Figure 2:**
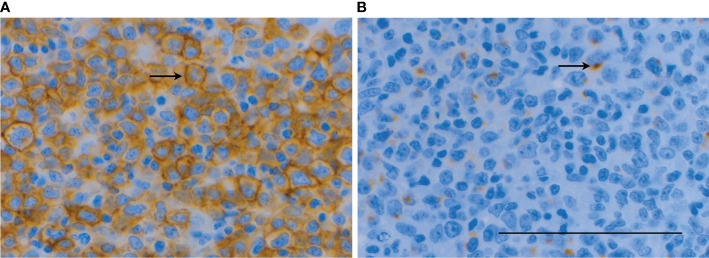
SSTR2 IHC **(A)** showed strong (score 3) immunopositivity on the cell membrane of malignant cells (arrows) in a patient with DLBCL of GCB-type (no. 35) with mild-to-moderate (score 1-2) cytoplasmic dot-like co-expression of CXCR4 **(B)** indicating to internalization of the receptor. Scale bar=100 µm.

Nearly half (46%, n=11) of the DLBCL patients had positive SSTR2 IHC with the expression being strong in 73% (n=8) of the cases (**Table 3**). SSTR2 expression was located mainly on the cell membrane of the malignant cells (n=10) (**Figure 2**). SSTR3 and SSTR5 were negative in DLBCL, except for two suspicious cases where SSTR3 was positive in one DLBCL patient (no. 30) who had mild staining in only 5% of the malignant cells and another patient (no. 40) who had mildly positive SSTR5 IHC, but strong background staining suggested that it might be a false positive.

**Table 3:** SSTR2, 3, 5 and CXCR4 IHC results in studied lymphoma subtypes.

Lymphoma	n=	score	SSTR2 n (%)	SSTR3 n (%)	SSTR5 n (%)	CXCR4 n (%)
**DLBCL**	24	negative	13 (54%)	23 (96%)	23 (96%)	9 (38%)
		mild	2 (8%)	1 (4%)		7 (29%)
		moderate	1 (4%)		1 (4%)	7 (29%)
		strong	8 (33%)			1 (4%)
**FL**	22	negative	10 (45%)			12 (55%)
		mild	5 (23%)			6 (27%)
		moderate	2 (9%)			3 (14%)
		strong	5 (23%)			1 (4%)
**HL**	18	negative	8 (44%)	11 (65%)	14 (78%)	4 (23%)
		mild	3 (17%)	4 (23%)	3 (17%)	6 (35%)
		moderate	2 (11%)	2 (12%)	1 (5%)	4 (23%)
		strong	5 (28%)			3 (18%)
**MCL**	20	negative	20 (100%)	20 (100%)	19 (95%)	17 (85%)
		positive			1 (5%)	3 (15%)
**PTCL**	10	negative	9 (90%)	10 (100%)	7 (70%)	7 (70%)
		positive	1 (10%)		3 (30%)	3 (30%)
**MALT**	9	negative	9 (100%)	9 (100%)	9 (100%)	9 (100%)

CXCR4 IHC was positive in 62% of the DLBCL patients but the staining was mostly mild or moderate. CXCR4 expression was cytoplasmic in all cases and had a specific dot-like pattern in 47% and a simultaneous expression on the cell membrane in 47% of the cases. Co-expression of SSTR2 and CXCR4 was present in 29% of the cases (n=7) where SSTR2 expression was typically strong accompanied with mild-to-moderate CXCR4 expression (n=4).

### Follicular Lymphoma

**Figure 3:**
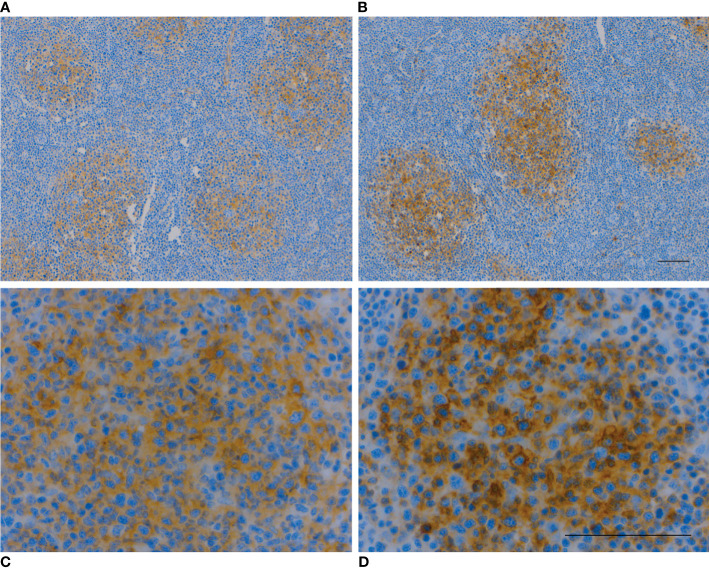
SSTR2 IHC **(A, C)** and CXCR4 IHC **(B, D)** were mildly-to-moderately positive in the cytoplasm and cell membrane in 70% of malignant B-cells in a patient with FL (no. 73). Follicular dendritic cells were also positive. Scale bar=100 µm.

Of all studied lymphoma subtypes patterns of receptor expression were closest to each other in DLBCL and FL. In line with this, SSTR2 immunopositivity was observed in 54% (n=12) of the FL patients (**Table 3**) with the expression being mostly membranous (n=7) or combined membranous and cytoplasmic (n=3). Two patients had SSTR2 expression only in the cytoplasm of the malignant cells. 45% (n=10) of the FL patients had positive CXCR4 IHC and the expression was predominantly membranous (n=8) with few cytoplasmic or combined cases (**Figure 3**). Nine patients (41%) showed co-expression of SSTR2 and CXCR4.

### Hodgkin Lymphoma

**Figure 4:**
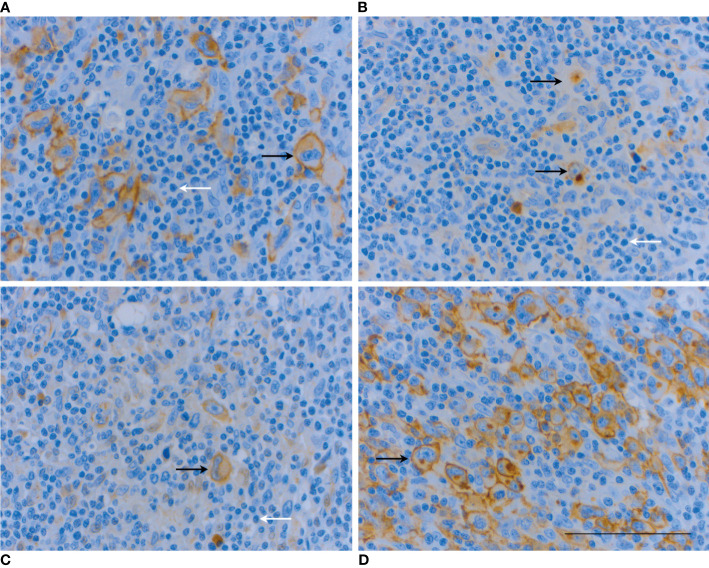
IHC analysis of a patient with HL mixed cellularity subtype (no. 50) shows co-expression of all studied receptors in various cell compartments. SSTR2 IHC **(A)** is strongly positive (score 3) on the cell membrane of Reed-Sternberg and Hodgkin cells (arrows). SSTR3 IHC **(B)** and SSTR5 IHC **(C)** are mildly positive (score 1) in the cytoplasm of the malignant cells (arrows). The most extensive receptor expression is with CXCR4 where IHC showed strong immunopositivity on the cell membrane and cytoplasm of 90% of the malignant cells **(D)**. Note weak or absent expression of all receptors in most non-neoplastic cells, including reactive small lymphocytes (white arrows). Scale bar=100 µm.

HL showed distinct and more heterogenous receptor profile compared to DLCBL and FL. We observed SSTR2, SSTR3, SSTR5 and CXCR4 immunopositivity in 56%, 35%, 22% and 76% of the cases, respectively (**Table 3**). The intensity of SSTR2 staining varied, but the majority of the SSTR2 positive patients had immunopositivity on the cell membrane of over 80% of the malignant Reed-Sternberg and Hodgkin cells. On the contrary, SSTR3 and SSTR5 expression – when present - was located in the cytoplasm of these malignant cells. (**Figure 4**) Fibrous bands characteristic of nodular sclerosis subtype of HL showed SSTR3 immunopositivity in 44% of cases but staining in connective tissues was seen in other lymphomas and tissues as well.

CXCR4 staining was typically both cytoplasmic and membranous. Homogenous cytoplasmic, homogenous membranous and dot-like cytoplasmic staining patterns were also observed. Co-expression of SSTR subtypes was evident in a total of three cases (**Figure 4**). Co-expression of SSTR subtypes and CXCR4 was seen in 65% of the HL patients, with SSTR2 being clearly the most common pair (n=8, 44%) for the chemokine receptor. One HL patient (no. 47) had unrepresentative SSTR3 and CXCR4 IHC which remained the only two assays classified as technical failures in the current study.

### Other Lymphomas

In comparison to DLBCL, FL and HL receptor expression in PTCL, MCL and MALT was uncommon and low. Only few positive findings were observed in PTCL (n=10) where 30% of the patients had positive SSTR5 and 30% had positive CXCR4 IHC. One PTCL patient (no. 90) co-expressed both SSTR5 and CXCR4. In stark contrast to DLBCL and FL, SSTR2 IHC was only mildly positive in one patient (no. 93), although 90% of the malignant cells stained positive. Interestingly, the only cytotoxic PTCL patient (no. 86) included in the study had strongly positive SSTR5 IHC in 70% of the malignant cells while other receptors were negative.

Out of 20 studied MCL patients only three (15%) showed any positive findings on IHC. One patient with blastoid variant (no. 2) had mild membranous CXCR4 expression. Another patient also with blastoid variant (no. 5) had mild cytoplasmic dot-like CXCR4 expression accompanied with mild cytoplasmic co-expression of SSTR5. Interestingly, both of these blastoid variants were found in the nasopharynx while the remaining two blastoid variants were nodal diseases and completely negative for receptor expression. Third patient with pleomorphic MCL variant (no. 20) had strong CXCR4 expression at the cell membrane of the malignant cells. SSTR2 and SSTR3 were negative in all MCL patients.

In this study all MALT lymphomas were negative for all SSTR receptors and CXCR4. Please see Supplementary Material available from the website for comprehensive data presentation.

### Receptor Expression in Non-Neoplastic Cells

Benign reactive lymphocytes were mainly negative in all stainings compared to malignant lymphoma cells, as can also be seen in figures. Strong staining in the background non-neoplastic cells was seen in some cases, i.e. SSTR3 was strongly positive in endothelial cells of veins. In addition, SSTR3 immunopositivity was observed in macrophages, mast cells and in connective tissue in fibroblasts. SSTR2 immunopositivity was seen consistently in macrophages, neutrophils, follicular dendritic cells and endothelial cells. SSTR5 positivity was noticed in endothelial cells and some macrophages and plasma cells. CXCR4 was expressed in some benign follicular cells and immunoblasts.

## Discussion

We undertook current study to characterize SSTR and CXCR4 expression in 103 patients with lymphoma. Recognizing their individual impact on lymphoma progression we were specifically interested in receptor co-expression which might assist in selection and timing of theranostic approach or in circumventing resistance to standard anti-lymphoma agents such as rituximab. We observed SSTR2 and CXCR4 immunopositivity in DLBCL, FL and HL in approximately half of the patients and co-expression of both receptors in 38% of the three lymphoma subtypes. Only in HL was co-expression of other SSTR receptors common while few cases of PTCL were positive for SSTR3 and to our surprise all 9 cases of MALT were negative for both SSTR and CXCR4 and similarly the majority of MCLs were receptor negative.

The role and clinical significance of SSTRs in lymphomas has long been under discussion (9, 11) and only few recent studies have added to this existing debate. In our previous pilot study of 21 patients, SSTR2 IHC was positive in malignant cells in one DLBCL patient and in all HL patients, and also all four patients with FL showed SSTR2 immunopositivity in neoplastic follicles with scattered positivity in the malignant B-cells (8). Tao et al. showed SSTR2a immunopositivity in the follicular dendritic cells in all 17 FLs (19). Recently, one pediatric HL case was reported to co-express mRNA for all five SSTR subtypes (SSTR1-5) (20) and in a small cohort of aggressive nasopharyngeal B-cell NHL, 40% of the 15 cases were SSTR2 positive (21). As a summary of these three and our own previous pilot study which all support current findings it is clear that SSTR2 and at least in HL also other SSTR subtypes are important for lymphoma growth but expression may be absent or low in a considerable number of cases.

Compared to SSTR, the role of CXCR4 has been studied more recently in lymphomas, especially in DLBCL, where CXCR4 upregulation has been shown to be associated with tumor cell dissemination, disease progression, and poor survival (14, 18, 22) and also with impaired response to rituximab treatment (23). Furthermore, CXCR4 antagonists have prevented disease progression and improved therapeutic response to rituximab treatment *in vitro* (16, 17). In the present study, 62% of DLBCL patients were positive on CXCR4 IHC which is in excellent coherence with a study by Xu et al. (2018) where 61% of the rituximab treated DLBCL patients had positive CXCR4 IHC (24). By contrast, Stollberg et al. (15) reported high CXCR4 expression and also less frequent SSTR expression in MALT type lymphomas, whereas our nine MALT patients showed completely negative IHC for all studied receptors. Two of our MALT patients (22%) had lymphoma of gastric origin which is in line with Stollberg et al. where 20% of patients had MALT of gastric origin. The antibody against CXCR4 in their study was the same although from different vendor and leaves us to speculate whether analytical issues rather than biology explain discrepant findings. In another study, MCL cells expressed high levels of functional CXCR4 (25), which was not the case in our study. In summary, we confirm CXCR4 expression in the more common subtypes DLBCL, FL and HL but advocate further investigation of this chemokine receptor in MCL and MALT where both pre-clinical and clinical studies are few.

There are several limitations to our study. First, rate of recruitment from recently treated patients was rather slow due to required informed consent which limited number of analyzed cases where initial goal was to include at least 20 patients from each lymphoma subtype. Due to slow accrual we included also relapsed and transformed diseases in our analysis but did not find any differences when compared to diagnostic tissue samples obtained pretreatment. Second, inclusion of several subtypes compelled us to refrain from statistical analysis of receptor expression and prognosis due to heterogeneity of cases. Comprehensive survival analysis on all patients as one group is not feasible since all selected lymphoma subtypes represent separate disease entities. Finally, as there is no standardized evaluation system for SSTR and CXCR4 expression at IHC in lymphomas, we had to develop our own system by adapting some of the varying scoring methods used in earlier studies. Immunoreactive score (IRS) by Remmele et Steigner (1987) (26) was not eventually used since our aim was to describe findings in lymphomas which are comprised from cell populations presenting heterogeneously in various subtypes. The IRS was originally developed for IHC analysis in breast cancer and is in our opinion too robust for current study. We acknowledge, however, that IRS has been successfully used in NETs (27) and was also adapted for lymphoma by Stollberg et al. (15).

Interestingly our analysis suggested that SSTR2 immunopositivity was associated with favorable prognosis and normal serum lactate dehydrogenase (LDH) level in DLBCL. SSTR5 expression on the other hand seemed to be linked to a more aggressive disease (data not shown). We must state these assumptions with caution since low number of cases prevented formal statistical analysis. Previously SSTR2 expression has been connected to better prognosis in NETs, pulmonary carcinoids and oligodendrogliomas (28–30). To shed light on prognostic significance of SSTR expression in lymphomas further studies with sufficient statistical power are warranted. Although the potential of CXCR4 as a biomarker has been recognized much later than that of SSTRs it is fair to say that expression of CXCR4 is implicated in metastatic potential, therapeutic resistance, and hostile microenvironment in many solid tumors and hematologic malignancies which include B-cell lymphomas (14). Therefore, it would be of high interest to study prognosis of lymphomas co-expressing SSTR2 and CXCR4 since the former seems to be a favorable and the latter an unfavorable biomarker.

In conclusion, SSTR and CXCR4 expression is heterogenous and varies considerably within different lymphoma subtypes. However, both SSTR2 and CXCR4 are commonly expressed in DLBCL, FL and HL rendering these lymphomas - when receptor positive - potential candidates for treatments targeting SSTR2/CXCR4. Even low receptor density could be beneficial in PRRT with 117Lu owing to the high radiation sensitivity of the majority of NHL and HL. Of note is that HL shows expression of SSTR3/5 in approximately quarter of the cases while DLBCL/FL show less consistent expression of SSTR3/5. Finally, survival of patients with lymphomas co-expressing SSTRs and CXCR4 should be studied to establish the prognostic role of these biomarkers in more detail.

## Data Availability Statement

The original contributions presented in the study are included in the article/**Supplementary Material**. Further inquiries can be directed to the corresponding author.

## Ethics Statement

The studies involving human participants were reviewed and approved by Ethics Committee of the Hospital District of Southwest Finland. The patients/participants provided their written informed consent to participate in this study. This study was also approved by Turku Clinical Research Centre and by the local university-based biobank (Auria Biobank).

## Author Contributions

All authors participated in designing the study. TJ was responsible for data gathering and co-operation with antibody supplier, private laboratory and Auria Biobank. SV analyzed all immunohistochemical stainings. TV performed statistical analysis. TJ wrote the final manuscript. SV, HM, and SJ modified the manuscript. All authors contributed to the article and approved the submitted version.

## Funding

This study was financially supported by Turku University Hospital research funds (EVO), Cancer Society of Finland and University of Turku.

## Conflict of Interest

The authors declare that the research was conducted in the absence of any commercial or financial relationships that could be construed as a potential conflict of interest.
