# Supplementary material for: Somatostatin Receptors and Chemokine Receptor CXCR4 in Lymphomas: A Histopathological Review of Six Lymphoma Subtypes
Source: Front Oncol. 2021 Jul 8;11:710900. doi: 10.3389/fonc.2021.710900 (PMC8299948; doi:10.3389/fonc.2021.710900)
Supplement: Supplementary file 1 [file Table_1.pdf]

SUPPLEMENTAL MATERIAL 1. SSTR2, 3, 5 and CXCR4 immunohistochemical stainings of 103 lymphoma patients.

				SSTR2		SSTR3		SSTR5		CXCR4	
Patient ID	Lymphoma subtype	Biopsy location	Biopsy type	Intensity	Percent	Intensity	Percent	Intensity	Percent	Intensity	Percent
N1	MCL	Bone marrow	Trephine	0		0		0		0	
N2	MCL (blastoid)	Nasopharynx	Surgical	0		0		0		1 (m)	50
N3	MCL	Lymph node (ingunal)	Surgical	0		0		0		0	
N4	MCL	Lymph node (ingunal)	Surgical	0		0		0		0	
N5	MCL (blastoid)	Nasopharynx	Surgical	0		0		1 (c)	30	1 (c dot)	20
N6	MCL	Lymph node (axillary)	Surgical	0		0		0		0	
N7	MCL	Bone marrow	Trephine	0		0		0		0	
N8	MCL (blastoid)	Lymph nore (cervical)	Surgical	0		0		0		0	
N9	MCL	Bone marrow	Trephine	0		0		0		0	
N10	MCL	Bone marrow	Trephine	0		0		0		0	
N11	MCL	Bone marrow	Trephine	0		0		0		0	
N12	MCL	Lymph node (inguinal)	Surgical	0		0		0		0	
N13	MCL	Lymph node (inguinal)	Surgical	0		0		0		0	
N14	MCL	Lymph node (axillary)	Surgical	0		0		0		0	
N15	MCL (blastoid)	Lymph node (inguinal)	Surgical	0		0		0		0	
N16	MCL	Lymph node	Core	0		0		0		0	
N17	MCL	Lymph nore (cervical)	Surgical	0		0		0		0	
N18	MCL	Lymph node (inguinal)	Surgical	0		0		0		0	
N19	MCL	Hypopharynx	Surgical	0		0		0		0	
N20	MCL (pleomorphic)	Lymph node (cecum)	Surgical	0		0		0		3 (m)	90
N21	DLBCL (ABC)	Lymph nore (cervical)	Surgical	0		0		0		1 (c dot)	20
N22	DLBCL (GCB)	Tonsil	Surgical	2 (m)	100	0		0		0	
N23	DLBCL (ABC)	Lymph node (axillary)	Surgical	0		0		0		1 (c dot)	10
N24	DLBCL (GCB)	Kidney	Core	0		0		0		2 (m/c)	50
N25	DLBCL (GCB)	Tonsil	Surgical	2 (m)	90	0		0		1 (c)	5
N26	DLBCL (GCB)	Lymph node (inguinal)	Surgical	3 (m)	100	0		0		1 (c)	10

N27	DLBCL (ABC)	Testis	Surgical	3 (m/c)	80	0		0		1 (c)	10
N28	DLBCL (GCB)	Lymph node (cervical)	Surgical	1 (c dot)	10	0		0		2 (m/c dot)	50
N29	DLBCL (ABC)	Upper jaw	Surgical	3 (m)	100	0		0		0	
N30	DLBCL (ABC)	Lymph node (axillary)	Surgical	0		1 (c dot)	5	0		2 (c dot)	50
N31	DLBCL (GCB)	Tonsil	Surgical	3 (m)	100	0		0		2 (m/c)	50
N32	DLBCL (ABC)	Mediastinum	Surgical	2 (m/c)	100	0		0		0	
N33	DLBCL (GCB)	Lymph node (cervical)	Surgical	0		0		0		0	
N34	DLBCL (GCB)	Lymph node (inguinal)	Surgical	0		0		0		2 (m/c)	70
N35	DLBCL (GCB)	Upper jaw	Surgical	3 (m)	90	0		0		1 (c dot)	20
N36	DLBCL (GCB)	Small intestine	Surgical	0		0		0		2 (c dot)	50
N37	DLBCL (GCB)	Lymph node jaw	Surgical	2 (m)	70	0		0		2 (m/c)	70
N38	DLBCL (GCB)	Spinal column	Surgical	1 (m)	20	0		0		0	
N39	DLBCL (ABC)	Lymph node (cervical)	Surgical	0		0		0		0	
N40	DLBCL (GCB)	Mesentery	Surgical	0		0		1 (c)	100	0	
N41	DLBCL (ABC)	Small intestine	Surgical	0		0		0		1 (m/c dot)	2
N42	DLBCL (T-cell predominance)	Lymph node (mesentery)	Surgical	0		0		0		2 (m/c)	80
N43	DLBCL (GCB)	Abdomen	Core	0		0		0		0	
N44	DLBCL (GCB)	Lymph node (cervical)	Surgical	0		0		0		0	
N45	HL nodular sclerosis	Skin	Surgical	0		0		1 (c)	50	1 (c)	40
N46	HL mixed cellularity	Lymph node (cervical)	Surgical	2 (m)	80	0		0		1 (m/c dot)	90
N47	HL nodular sclerosis	Lymph node (cervical)	Surgical	2 (m)	80			0			
N48	HL nodular sclerosis	Lymph node (cervical)	Surgical	0		0		0		0	
N49	HL mixed cellularity	Lymph node (cervical)	Surgical	1 (m)	70	1 (m/c)	50	1 (c)	50	0	
N50	HL mixed cellularity	Lymph node (axillary)	Surgical	2 (m/c)	80	1 (c)	30	1 (c)	30	3 (m/c)	90
N51	NLPHL	Lymph node (axillary)	Surgical	0		0		1 (c)	80	0	
N52	HL mixed cellularity	Lymph node (cervical)	Surgical	0		0		0		2 (m/c dot)	90
N53	HL nodular sclerosis	Lymph node (cervical)	Surgical	0		1 (c)	80	0		0	
N54	NLHPL	Lymph node (axillary)	Surgical	1 (m)	50	0		0		1 (m)	50
N55	HL mixed cellularity	Colon	Surgical	3 (m)	80	0		0		2 (m/c)	80

N56	HL nodular sclerosis	Lymph node (inguinal)	Surgical	2 (m)	80	0		0		2 (m/c)	60
N57	HL nodular sclerosis	Lymph node mediastinum	Surgical	1 (m)	90	0		0		1 (m)	40
N58	HL nodular sclerosis	Lymph node (axillary)	Surgical	1 (m)	25	1 (c)	25	0		2 (m/c)	70
N59	HL nodular sclerosis	Lymph node (cervical)	Surgical	1 (m)	80	0		0		1 (m)	70
N60	HL mixed cellularity	Spleen	Surgical	0		2 (c)	40	0		1 (m/c)	50
N61	CHL NOS	Bone	Core	0		1 (c dot)	60	0		1 (c dot)	60
N62	HL nodular sclerosis	Lymph node (cervical)	Surgical	0		0		0		2 (m/c)	70
N63	Follicular lymphoma	Abdomen	Core	2 (m)	100	0		0		1 (m)	5
N64	Follicular lymphoma	Lymph node (axillary)	Surgical	1 (m)	100	0		0		1 (m)	20
N65	Follicular lymphoma	Lymph node (cervical)	Surgical	2 (m)	100	0		0		0	
N66	Follicular lymphoma	Lymph node (mesenterium)	Surgical	2 (m/c)	100	0		0		1 (m/c)	40
N67	Follicular lymphoma	Lymph node (cervical)	Surgical	1 (m)	50	0		0		0	
N68	Follicular lymphoma	Lymph node (axillary)	Surgical	0		0		0		2 (m/c)	80
N69	Follicular lymphoma	Lymph node (cervical)	Surgical	1 (c)	50	0		0		2 (m)	50
N70	Follicular lymphoma	Lymph node (mesenterium)	Surgical	0		0		0		1 (c)	40
N71	Follicular lymphoma	Lymph node (mesenterium)	Surgical	1 (m)	20	0		0		1 (m)	20
N72	Follicular lymphoma	Lymph node (abdomen)	Surgical	1 (c)	50	0		0		1 (c)	50
N73	Follicular lymphoma	Lymph node (abdomen)	Surgical	1 (m/c)	70	0		0		2 (m/c)	70

N74	Follicular lymphoma	Lymph node (cervical)	Surgical	0		0		0		0	
N75	Follicular lymphoma	Lymph node (axillary)	Surgical	0		0		0		0	
N76	Follicular lymphoma	Lymph node (abdomen)	Core	0		0		0		0	
N77	Follicular lymphoma	Lymph node (cervical)	Surgical	0		0		0		0	
N78	Follicular lymphoma	Tonsil	Surgical	0		0		0		0	
N79	Follicular lymphoma	Lymph node (cervical)	Surgical	2 (m/c)	80	0		0		0	
N80	Follicular lymphoma	Lymph node (cervical)	Surgical	0		0		0		0	
N81	Follicular lymphoma	Lymph node (cervical)	Surgical	0		0		0		0	
N82	Follicular lymphoma	Lymph node (cervical)	Surgical	3 (m)		0		0		2 (m)	25
N83	Follicular lymphoma	Lymph node (inguinal)	Surgical	2 (m)		0		0		0	70
N84	Follicular lymphoma	Lymph node (cervical)	Surgical	0		0		0		0	
N85	PTCL	Lymph node (axillary)	Surgical	0		0		0		0	
N86	PTCL (cytotoxic)	Nose	Surgical	0		0		3 (m)	70	0	
N87	PTCL	Lymph node (inguinal)	Core	0		0		0		0	
N88	PTCL (Lennert)	Lymph node (axillary)	Surgical	0		0		0		0	
N89	PTCL	Lymph node (leg)	Surgical	0		0		0		0	
N90	PTCL	Sternum	Core	0		0		1 (c)	50	1 (c dot)	15
N91	PTCL	Lymph node (cervical)	Surgical	0		0		0		0	

N92	ALCL (breast-implant associated)	Breast	Surgical	0		0		0		2 (m)	50
N93	ALCL	Skin	Surgical	1 (m)	90	0		2 (c)	90	0	
N94	ALCL	Bone marrow	Trephine	0		0		0		1 (m/c)	50
N95	Extranodal MALT	Lymph node (inguinal)	Surgical	0		0		0		0	
N96	Extranodal MALT	Submandibular gland	Surgical	0		0		0		0	
N97	Gastric MALT	Stomach	Surgical	0		0		0		0	
N98	Extranodal MALT	Parotid gland	Surgical	0		0		0		0	
N99	Extranodal MALT	Lymph node (cheek)	Surgical	0		0		0		0	
N100	Extranodal MALT	Conjunctiva	Surgical	0		0		0		0	
N101	Extranodal MALT	Lymph node (cervical)	Surgical	0		0		0		0	
N102	Extranodal MALT	Bone marrow	Trephine	0		0		0		0	
N103	Gastric MALT	Stomach	Surgical	0		0		0		0	

MCL=mantle cell lymphoma, DLBCL=diffuse large B-cell lymphoma, ABC=activated B-cell, GCB=germinal center B-cell, HL=Hodgkin lymphoma, CHL=classic Hodgkin lymphoma, NOS=not otherwise specified, NLPHL=nodular lymphocyte-predominant Hodgkin lymphoma, PTCL=peripheral T-cell lymphoma, ALCL=anaplastic large B-cell lymphoma, MALT=mucosa-associated lymphoid tissue lymphoma.

Staining intensity is scored as no staining (0), mild (1), moderate (2) or strong (3).
Percent is presented as how many percent of the malignant cells were stained positive.

(m)=membranous staining in the malignant cells
(c)=cytoplasmic staining in the malignant cells
(c dot)=cytoplasmic dot-like staining in the malignant cells
